# Risk factors for ocular hypertension after intravitreal dexamethasone implantation in diabetic macular edema

**DOI:** 10.1038/s41598-020-70833-1

**Published:** 2020-08-13

**Authors:** Moon Young Choi, Jin-woo Kwon

**Affiliations:** 1 Department of Ophthalmology, St. Vincent’s Hospital, College of Medicine, The Catholic University of Korea, Seoul, Korea; 2 Department of Ophthalmology and Visual Science, St. Vincent’s Hospital, Jungbu-daero 93, Paldal-gu, Suwon, 16247 Korea

**Keywords:** Eye diseases, Retinal diseases

## Abstract

We designed a retrospective observational study to identify factors associated with ocular hypertension [OHTN, defined as intraocular pressure (IOP) > 25 mmHg] after intravitreal dexamethasone (IVD) implantation in diabetic macular edema (DME) patients. We measured IOP monthly after placement of an IVD implant, and identified the trend of IOP, incidence of OHTN, and its associated systemic or ocular factors. On average, IOP was highest at 2 months after IVD implantation. Of 84 DME patients who received an IVD implant, 3 (3.57%) presented with an IOP ≥ 25 mmHg at 1 month after implantation, 6 (7.14%) at 2 months, and 2 (2.38%) at 3 months. Compared with the non-OHTN group, the OHTN group had significantly shorter axial lengths and were younger. Logistic regression analysis revealed that axial length < 23.00 mm and age < 57 years were associated with OHTN occurrence. Patients whose IOP was elevated > 30% or ≥ 20 mmHg at 1 month post-implantation, subsequently developed OHTN with statistical significance. In conclusion, shorter axial length and younger age were associated with OHTN occurrence after IVD implantation. Additionally, identifying levels or trends in IOP at 1 month after the IVD procedure may be helpful in predicting subsequent OHTN occurrence.

## Introduction

Diabetic macular edema (DME) is one of the main causes of visual impairment in diabetes patients^1,2^. Although the exact mechanism has yet to be determined, one of the early signs of DME pathogenesis is damage to the blood–retina barrier, which occurs in association with metabolic changes, ischemia, and inflammation^3,4^.

Based on fundamental studies on vascular endothelial growth factor (VEGF) and randomized controlled trials of anti-VEGF agents, intravitreal injection of anti-VEGF agents has become the main treatment option for DME^5–8^. Recently, micronized dexamethasone in a biodegradable copolymer has been introduced as another treatment option for refractory or chronic DME^9–11^. Intravitreal dexamethasone (IVD) implantation makes it easier to control inflammation, which plays an important role in DME pathogenesis^12^. Several studies have reported that IVD implantation is effective for restoring macular structure, thus contributing to improved visual function in DME patients^11,13,14^. However, IVD implantation has potential complications, such as cataract formation and ocular hypertension (OHTN)^13,15^. Diabetes patients are more susceptible to glaucoma as a result of elevated intraocular pressure (IOP)^16,17^. Additionally, recent studies have reported that even eyes with no apparent diabetic retinopathy may develop glaucoma-related changes, including thinning of the retinal nerve fiber layer or macula ganglion cell-inner plexiform layer^18,19^. Thus, appropriate management and interventions are needed to prevent irreversible visual impairment in OHTN cases.

Although some studies have reported the prevalence of OHTN after IVD, few have examined the risk factors or precise time of occurrence of OHTN^20,21^. Thus, we examined DME patients on a monthly basis after IVD implantation to identify the risk factors for OHTN and the incidence thereof.

## Results

**Figure 1:**
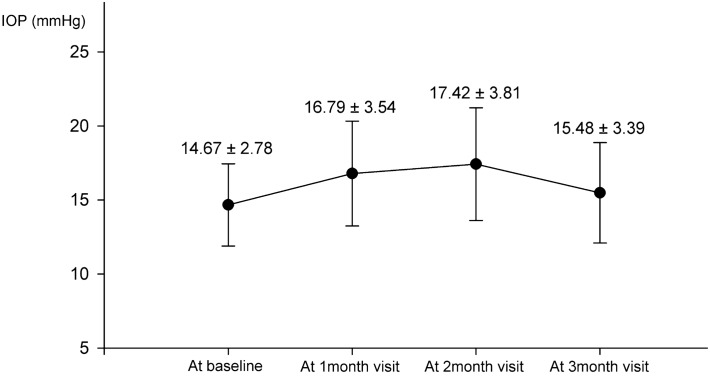
Changes in average intraocular pressure (IOP) after the intravitreal dexamethasone implant procedure. The IOP was measured at baseline, and at 1, 2, and 3 months after the procedure. The highest average IOP occurred at 2 months after the procedure but had decreased by the 3-month check-up.

The average age of the 84 enrolled patients was 57.63 ± 7.81 years. There were 35 males and 49 females. The average duration of diabetes was 9.70 ± 6.96 years. The baseline central subfield thickness (CST) was 494.19 ± 129.33 µm, and the thinnest CST after dexamethasone implantation was 275.12 ± 47.04 µm. The average interval between treatment and DME recurrence was 3.93 ± 0.90 months. Of the 84 DME patients who received an IVD implant, 11 (13.10%) developed OHTN (IOP ≥ 25 mmHg), and 9 (10.71%) showed IOP increase ≥ 10 mmHg compared with baseline IOP within 3 months after the procedure. Postoperatively, OHTN occurred in three patients (3.57%) at 1 month, in six (7.14%) at 2 months, and in two (2.38%) at 3 months. After excluding patients who had been diagnosed with OHTN at a previous visit, average IOPs were 14.67 ± 2.78 mmHg at baseline, 16.79 ± 3.54 mmHg at 1 month, 17.42 ± 3.81 mmHg at 2 months, and 15.48 ± 3.39 mmHg at 3 months after the IVD procedure. The maximum value of IOP was measured at 2 months after implant, and the mean value of IOP increments was 2.77 ± 3.85 mmHg compared with baseline IOP (Fig. 1).

The OHTN group had shorter axial lengths and were younger compared with the non- OHTN group (p = 0,012 and p = 0.004, respectively). However, other factors, including baseline IOP (non-OHTN group: 14.52 ± 2.80 mmHg, OHTN group: 15.64 ± 2.54, p = 0.216), a previous history of vitrectomy and lens status, did not differ between the two groups. Additionally, in terms of responsiveness, the CST reduction, thinnest CST during follow-up, and action duration of dexamethasone showed no significant differences between the two groups (Table 1).

**Table 1:** Demographic and clinical characteristics of DME patients diagnosed with OHTN after IVD implantation.

		Non- OHTN group (n = 73)	OHTN group (n = 11)	P-value
**Systemic factors**				
Sex (male:female)		29:44	6:5	0.548
Age (years)		58.58 ± 7.12	51.36 ± 9.57	0.004
Duration of diabetes (years)		10.00 [3.00;13.00]	8.00 [5.50;12.00]	0.963
HbA1c (%)		7.20 [6.40;7.70]	7.50 [6.70;7.50]	0.715
**Ocular factors**				
Baseline IOP (mmHg)		14.52 ± 2.80	15.64 ± 2.54	0.216
Maximum IOP increase (mmHg)		3.0 [2.0;5.0]	11.0 [8.5;13.0]	< 0.001
Axial length (mm)		23.33 [22.94;23.71]	22.87 [22.56;23.02]	0.012
Pseudophakic eyes		26 (35.62%)	2 (18.18%)	0.423
Vitrectomized eyes		11 (15.07%)	2 (18.18%)	1.000
DMR stage	Moderate NPDR	9 (12.33%)	1 (9.09%)	0.805
	Severe NPDR	11 (15.07%)	1 (9.09%)	
	PDR	53 (72.60%)	9 (81.82%)	
**OCT findings**				
Baseline CST (µm)		470.00 [381.00;591.00]	434.00 [375.50;623.00]	0.730
Thinnest CST after implantation		273.00 [242.00;290.00]	263.00 [233.00;283.50]	0.490
Maximum CST reduction		211.00 [100.00;321.00]	165.00 [119.50;276.50]	0.811
DME recurrence interval (months)		4.00 [3.00;4.00]	4.00 [3.00;4.50]	0.714

Values are expressed as mean ± SD or median and interquartile range, as appropriate.

*DME* diabetic macular edema, *OHTN* ocular hypertension, *IVD* intravitreal dexamethasone, *HbA1c* glycated hemoglobin, *IOP* intraocular pressure, *DMR* DM retinopathy, *NPDR* non-proliferative diabetic retinopathy, *PDR* proliferative diabetic retinopathy, *CST* central subfield thickness.

There were thirteen patients who had a history of vitrectomy. Between vitrectomized eyes and non-vitrectomized eyes, there was no significant difference in the IOP increases after IVD implantation (vitrectomized eyes: 3.15 ± 2.58 mmHg vs. non-vitrectomized eyes: 4.30 ± 3.65, p = 0.280). Not only the changes of IOP, but also the effects of dexamethasone implants were not different between them. The thinnest CST after IVD implantation (vitrectomized eyes: 267.23 ± 36.95 µm vs. non-vitrectomized eyes: 276.56 ± 48.75 µm, p = 0.578) and action duration of dexamethasone implant (vitrectomized eyes: 3.77 ± 0.60 months vs. non-vitrectomized eyes: 3.96 ± 0.95 months, p = 0.689) were not significantly different.

In the logistic regression analysis to identify factors associated with OHTN development after IVD implantation, axial length < 23.00 mm^22,23^ and age < 57 years (the median value of age in enrolled DME patients) were statistically significant (OR: 4.17, p = 0.042; and OR: 4.65, p = 0.038, respectively; Table 2).

**Table 2:** Logistic regression analysis of the factors associated with OHTN after IVD implantation in DME patients.

	Category	N (%)	Univariate		Multivariate	
			OR (95%CI)	P	OR (95%CI)	P
Sex	Female	49 (58.33%)	Reference			
	Male	35 (41.67%)	1.82 (0.50, 6.85)	0.357		
Age (years)	≥ 57	50 (59.52%)	Reference		Reference	
	< 57	34 (40.48%)	4.82 (1.27, 23.48)	0.029	4.65 (1.18, 23.37)	0.038
DMR stage	NPDR	22 (26.19%)	Reference			
	PDR	62 (73.81%)	1.70 (0.39, 11.77)	0.521		
Duration of diabetes (years)	< 10	42 (50.00%)	Reference			
	≥ 10	42 (50.00%)	0.81 (0.22, 2.92)	0.747		
Baseline IOP (mmHg)	≤ 15	32 (38.10%)	Reference			
	> 15	52 (61.90%)	1.42 (0.38, 5.15)	0.591		
Axial length (mm)	≥ 23.00	56 (66.67%)	Reference		Reference	
	< 23.00	28(33.33%)	4.33 (1.18, 18.03)	0.031	4.17 (1.09, 18.10)	0.042
Vitrectomized eye	Yes	13 (15.48%)	Reference			
	No	71 (84.52%)	1.25 (0.18, 5.74)	0.791		
Pseudophakic eye	Yes	28 (33.33%)	Reference			
	No	56 (66.67%)	0.40 (0.05, 1.71)	0.266		
Baseline CST (µm)	≤ 400	27 (32.14%)	Reference			
	> 400	57 (67.86%)	0.81 (0.22, 2.92)	0.748		

*OHTN* ocular hypertension, *IVD* intravitreal dexamethasone, *DME* diabetic macular edema, *OR* odds ratio, *CI* confidence interval, *DMR* DM retinopathy, *IOP* intraocular pressure, *CST* central subfield thickness.

After excluding three patients who had OHTN at 1 month after IVD, of nine patients exhibiting an IOP increase of over 30% at 1 month after the procedure, five (55.56%) subsequently developed OHTN (OR: 28.75, p < 0.0.01). Of 15 patients with an IOP ≥ 20 mmHg at 1 month after IVD implantation, six (40.00%) subsequently developed OHTN (OR: 21.33, p < 0.001).

## Discussion

In this retrospective study, which was performed to identify the risk factors for OHTN after IVD implantation, we found that younger age and shorter axial length were associated with OHTN occurrence. The IOP at 1 month after IVD implantation may be a significant predictor of subsequent OHTN development.

The mechanism of steroid-induced glaucoma (SIG) is to increase aqueous outflow resistance. Steroids can affect the microstructure of the trabecular meshwork (TM) by increasing the deposition of substances in the TM and decreasing the breakdown of substances through inhibition of endothelial cell phagocytosis^24–26^. The risk factors for SIG may differ depending on the administration route and steroid formulation^27^. The risk factors for SIG when using topical steroid eye-drops include pre-existing primary open-angle glaucoma, glaucoma suspect, young or old age, type I diabetes, and high myopia^27,28^. Intravitreal triamcinolone acetonide has additional risk factors, including pseudophakic eyes and previously vitrectomized eyes^29^. Recently, the SAFODEX study, which determined the incidence of OHTN after IVD implantation, also reported age under 60 years, male sex, preexisting glaucoma, and myopia with an axial length over 25 mm to be risk factors for the disease; however, previous vitrectomy and lens status were not associated with OHTN^21^. Another recent study reported that OHTN after IVD implantation was associated with the position of the implant in the pars plana region^30^. Additionally, SAFODEX study revealed that repeated IVD resulted in more OHTN^21^. However, another study reported that multiple IVD did not increase the frequency of IOP spikes beyond 30 mmHg^31^.

While a previous study reported high myopia to be a risk factor for OHTN after IVD implantation^21^, we found that a short axial length may represent an additional risk factor for OHTN. The incidence of myopia in the elderly is low in Korea and axial myopia protects against diabetic retinopathy; accordingly, all of the patients in our study had an axial length of less than 25 mm^32,33^. A short axial length is a risk factor for angle-closure glaucoma^34,35^. Although we did not suggest this in the Results section, the average increase in IOP was significantly greater in phakic eyes than in pseudophakic eyes (4.57 ± 4.31 vs. 2.71 ± 1.51 mmHg, p = 0.023). Taken together, these results imply that cataracts in diabetic patients may play a role in increased IOP, especially in those with short axial length, via the same mechanism through which angle-closure glaucoma occurs^36^. Further investigations are required to determine whether change of lens extenuates increased IOP after IVD implantation in patients with relatively short axial length.

In our study, although the average IOP value was obtained after excluding patients with OHTN at previous visits, IOP levels were higher than reported in other studies, as was OHTN prevalence. A recent study reported an average IOP at baseline of 13.45 mmHg, which increased to 16.85 mmHg at 2 months after IVD implantation; in addition, the prevalence of OHTN was 10.6%^20^. The SAFODEX study reported a prevalence of IOP ≥ 25 mmHg after the first IVD implantation of 11.4%^21^. We infer that differences among patient populations, including in terms of the proportion with relatively short axial lengths or relatively high baseline IOPs, could affect the results regarding IOP and OHTN prevalence.

To date, most studies have focused on IOP changes or OHTN prevalence after IVD implantation; however, the relationship between IOP trends and subsequent OHTN occurrence has not been evaluated^13,20,21^. Our study showed that IOP level or a change in IOP from the previous visit may be a significant factor for predicting subsequent OHTN. Based on our results, patients whose IOP increases > 30% or who have an IOP ≥ 20 mmHg at the 1-month visit after IVD implantation may be at significant risk of developing OHTN. Thus, checking IOP at 1 month after IVD implantation is important in the management of IVD patients.

In our study, thirteen patients had a history of vitrectomy. All of these patients had taken vitrectomy due to complications of diabetic retinopathy; ten for vitreous hemorrhage, and 3 for tractional membrane inducing visual disturbance. Between vitrectomized eyes and non-vitrectomized eyes, there were no significant differences in the maximum IOP increases or the thinnest CST after IVD implantation, and action duration of dexamethasone implant.

This study had several limitations. First, the sample size was relatively small; moreover, we did not analyze optical coherence tomography angiography findings or aqueous biomarkers. In addition, additional IVD injection was performed in patients who showed recurrence of center-involved DME, and the follow-up period was relatively short for evaluating long-term trends in IOP. To identify the effect of a short axial length on IOP, measurement of anterior chamber depth and gonioscopy or anterior segment OCT examinations should have been performed pre- and postoperatively. We are planning a follow-up prospective study to address these limitations.

In conclusion, age < 57 years and axial length < 23.00 mm were associated with the occurrence of OHTN after IVD implantation. Additionally, measuring IOP at 1 month after IVD implantation may be helpful for predicting subsequent OHTN development.

## Methods

The study protocol adhered to the tenets of the Declaration of Helsinki, and was approved by the Institutional Review Board and Ethics Committee of the Catholic University of Korea (protocol number: VC19RASI0280). The requirement for informed consent was waived because this study involved a review of patient records.

### Study population

We enrolled DME patients with a CST > 300 µm who presented to our center between 2016 and 2019 and received their first IVD implant. All of the participants were at least 20 years of age and had type II diabetes. We excluded patients diagnosed with uveitis, glaucoma, glaucoma suspect, retinal degeneration, or macular edema attributable to other causes.

### Study design

In addition to measurement of glycated hemoglobin levels, all patients underwent full ophthalmic examinations, including measurement of best-corrected visual acuity (BCVA) and IOP, as well as a dilated fundus examination. Macular thickness was measured by OCT (Cirrus High-Definition OCT; Carl Zeiss Meditec, Dublin, CA, USA). Ozurdex dexamethasone implants (Allergan, Irvine, CA, USA) were used in all patients, who were monitored at monthly intervals. We performed a dilated-fundus examination and evaluated the BCVA and CST at each visit up to 6 months after IVD implantation. For there are some patients who need additional treatments after 3 months after IVD implantation, we analyzed all parameters until center-involved DME recurrence (CST > 300 μm). Any adverse events were also recorded at each visit.

### IOP measurements

All patients underwent IOP measurement at baseline and at every month after IVD implantation. The IOP was checked using Goldmann applanation tonometer. We measured IOP up to 6 months after IVD implantation, and OHTN was defined as IOP of at least 25 mmHg^15,37^.

### Statistical analyses

All statistical analyses were performed using SPSS statistical software for Windows (version 21.0; IBM Corp., Armonk, NY, USA). The Mann–Whitney U-test or t-test was used to compare the OHTN group with the non-OHTN group. The chi-squared test or Fisher’s exact test was applied to compare parameters. Factors associated with OHTN occurrence were identified using logistic regression analysis. The level of statistical significance was set at p < 0.05 in all analyses.

## Data Availability

The datasets generated during and/or analyzed during the current study are available from the corresponding author on reasonable request.

## References

[1] Sivaprasad S, Gupta B, Crosby-Nwaobi R, Evans J (2012). Prevalence of diabetic retinopathy in various ethnic groups: A worldwide perspective. Surv. Ophthalmol..

[2] VanderBeek BL, Shah N, Parikh PC, Ma L (2016). Trends in the care of diabetic macular edema: Analysis of a national cohort. PLoS ONE.

[3] Das A, McGuire PG, Rangasamy S (2015). Diabetic macular edema: Pathophysiology and novel therapeutic targets. Ophthalmology.

[4] Tang J, Kern TS (2011). Inflammation in diabetic retinopathy. Prog. Retin. Eye Res..

[5] Wells JA (2015). Aflibercept, bevacizumab, or ranibizumab for diabetic macular edema. N. Engl. J. Med..

[6] Aiello LP (1994). Vascular endothelial growth factor in ocular fluid of patients with diabetic retinopathy and other retinal disorders. N. Engl. J. Med..

[7] Nguyen QD (2012). Ranibizumab for diabetic macular edema: results from 2 phase III randomized trials: RISE and RIDE. Ophthalmology.

[8] Network DRCR (2015). Aflibercept, bevacizumab, or ranibizumab for diabetic macular edema. N. Engl. J. Med..

[9] Unsal E, Eltutar K, Sultan P, Erkul SO, Osmanbasoglu OA (2017). Efficacy and safety of intravitreal dexamethasone implants for treatment of refractory diabetic macular edema. Korean J. Ophthalmol..

[10] Ramu J (2015). A randomized clinical trial comparing fixed vs pro-re-nata dosing of Ozurdex in refractory diabetic macular oedema (OZDRY study). Eye.

[11] Pacella F (2016). An eighteen-month follow-up study on the effects of intravitreal dexamethasone implant in diabetic macular edema refractory to anti-VEGF therapy. Int. J. Ophthalmol..

[12] Kuno N, Fujii S (2010). Biodegradable intraocular therapies for retinal disorders: Progress to date. Drugs Aging.

[13] Boyer DS (2014). Three-year, randomized, sham-controlled trial of dexamethasone intravitreal implant in patients with diabetic macular edema. Ophthalmology.

[14] Gillies MC (2014). A randomized clinical trial of intravitreal bevacizumab versus intravitreal dexamethasone for diabetic macular edema: The BEVORDEX study. Ophthalmology.

[15] Rajesh B (2020). Safety of 6000 intravitreal dexamethasone implants. Br. J. Ophthalmol..

[16] Klein BE, Klein R, Jensen SC (1994). Open-angle glaucoma and older-onset diabetes: The Beaver Dam eye Study. Ophthalmology.

[17] Zhou M, Wang W, Huang W, Zhang X (2014). Diabetes mellitus as a risk factor for open-angle glaucoma: A systematic review and meta-analysis. PLoS ONE.

[18] Oshitari T, Hanawa K, Adachi-Usami E (2009). Changes of macular and RNFL thicknesses measured by Stratus OCT in patients with early stage diabetes. Eye.

[19] Choi JA (2017). Early inner retinal thinning and cardiovascular autonomic dysfunction in type 2 diabetes. PLoS ONE.

[20] Choi W (2019). Intraocular pressure change after injection of intravitreal dexamethasone (Ozurdex) implant in Korean patients. Br. J. Ophthalmol..

[21] Malcles A (2017). Safety of intravitreal dexamethasone implant (OZURDEX): The SAFODEX study. Incidence and risk factors of ocular hypertension. Retina.

[22] Arora KS, Jefferys JL, Maul EA, Quigley HAJIO, Science V (2012). Choroidal thickness increase is different among angle-closure versus open-angle eyes but does not explain IOP rise after water drinking. IOVS.

[23] Kobayashi H, Kobayashi K, Okinami S (2002). Macular hole and myopic refraction. Br. J. Ophthalmol..

[24] Clark AF (2005). Dexamethasone alters F-actin architecture and promotes cross-linked actin network formation in human trabecular meshwork tissue. Cell Motil. Cytoskeleton..

[25] Yue BY (1996). The extracellular matrix and its modulation in the trabecular meshwork. Surv. Ophthalmol..

[26] Filla MS, Schwinn MK, Nosie AK, Clark RW, Peters DM (2011). Dexamethasone-associated cross-linked actin network formation in human trabecular meshwork cells involves beta3 integrin signaling. Invest. Ophthalmol. Vis. Sci..

[27] Jones R, Rhee D (2006). Corticosteroid-induced ocular hypertension and glaucoma: A brief review and update of the literature. Clin. Opin. Opthamol..

[28] Chang DF, Tan JJ, Tripodis YJ, Surgery R (2011). Risk factors for steroid response among cataract patients. J. Cataract. Refract. Surg..

[29] Vedantham VJ (2005). Intraocular pressure rise after intravitreal triamcinolone. Am. J. Opthamol..

[30] Sudhalkar A, Kodjikian L, Chhablani J, Bhojwani D, Vasavada A (2018). Intraocular dexamethasone implant position in situ and ocular hypertension. Retina.

[31] Bahadorani S (2018). The effects of repeated Ozurdex injections on ocular hypertension. Clin. Ophthalmol. (Auck, NZ).

[32] Choi JA, Han K, Park YM, Park CK (2014). Age-related association of refractive error with intraocular pressure in the Korea National Health and Nutrition Examination Survey. PLoS ONE.

[33] Lim LS (2010). Are myopic eyes less likely to have diabetic retinopathy?. Ophthalmology.

[34] Oh WH, Kim BG, Kyung H, Lee JH (2019). Primary angle-closure glaucoma with normal intraocular pressure at the first visit: Its prevalence and ocular characteristics. J. Glaucoma..

[35] Varma DK, Kletke SN, Rai AS, Ahmed IIK (2017). Proportion of undetected narrow angles or angle closure in cataract surgery referrals. Can. J. Ophthalmol..

[36] Jacobi PC, Dietlein TS, Lüke C, Engels B, Krieglstein GKJO (2002). Primary phacoemulsification and intraocular lens implantation for acute angle-closure glaucoma. Opthamology.

[37] Guigou S (2014). Multicenter Ozurdex(R) assessment for diabetic macular edema: MOZART study. J. Fr. Ophtalmol..

